# Assessment of Theory of Mind in Psychopathology: a Scoping Review

**DOI:** 10.1192/j.eurpsy.2023.2127

**Published:** 2023-07-19

**Authors:** P. de la Higuera-Gonzalez, A. Galvez-Merlin, E. Rodríguez-Toscano, J. Andreo-Jover, T. Lopez-Soto, A. de la Torre-Luque

**Affiliations:** 1 Department of Personality, Assessment and Clinical Psychology, Faculty of Psychology, Universidad Complutense de Madrid; 2 Health Research Institute, Hospital Clínico San Carlos (IdISSC); 3 Department of Legal Medicine, Psychiatry and Pathology, Faculty of Medicine; 4 Department of Experimental Psychology, Cognitive Processes and Speech Therapy, Universidad Complutense de Madrid; 5 Institute of Psychiatry and Mental Health, Hospital Gregorio Marañon; 6 Universidad Autónoma de Madrid; 7 Instituto de Investigación del Hospital Universitario La Paz (IdiPAZ), Madrid; 8 Universidad de Sevilla, Sevilla; 9 Biomedical Research Networking Consortium for Mental Health (CIBERSAM ISCIII), Madrid, Spain

## Abstract

**Introduction:**

Theory of Mind (ToM) is defined as the cognitive ability that infers other’s mental states (Premack & Woodruff. J Behav Brain Sci 1978; 1 515-526).The interest in the study of ToM distinguishing its affective and cognitive components has been growing. Its study in psychopathology has been evolved from its original studies in autism spectrum disorders (ASD), schizophrenia (SCZ) and, borderline personality disorder (BPD), to other mental disorders like major depressive disorder (MDD), bipolar disorder (BP), anorexia nervosa (AN) and social anxiety disorder (SAD).

**Objectives:**

1) review the most commonly used instruments for ToM assessment; 2) to compile the evidence on ToM deficits across mental disorders. For both objectives, target disorders are previously mentioned.

**Methods:**

The search was carried out on the PubMed, PsycInfo and Scopus databases, using the terms “Theory of mind”, “Mentalization” and the previously mentioned mental disorders and pertinent thesaurus. Articles in English, published since 2010 were considered. A 2-step strategy (first, article screening and full reading) was followed to select articles of interest.

**Results:**

Reading the Mind in the Eyes (Baron-Cohen et al., J Child Psychol Psychiatry 2001; 42 241-251) and Movie for the Assessment of Social Cognition (Dziobeck et al. J Autism Dev Disord 2006; 36 623-636) were the most commonly used tasks to assess ToM. Regarding mental disorders, studies showed deficits in cognitive and affective ToM skills in ASD, SCZ, BPD, MDD and BP. Hypomentalization was mainly observed in ASD and MDD, while BPD and SCZ were featured by errors associated with hypermentalization. Studies in AN and SAD are scarce, but they mainly highlight a cognitive ToM deficit, with hypomentalization in AN and hypermentalization in ASD. In all of them, depressive symptomatology seems to be a critical moderator of ToM performance.

**Conclusions:**

Although ToM impairments are well described for some mental disorders, more research is needed to reach solid conclusions for others. The use of different and heterogeneous ToM assessment instruments can strongly influence the results of studies. The study of ToM is essential to gain a better understanding of the diseases and to develop effective treatments targeting specific ToM deficits.

**Disclosure of Interest:**

None Declared

